# Enhancing the Performance of the Photonic Integrated Sensing System by Applying Frequency Interrogation

**DOI:** 10.3390/nano13010193

**Published:** 2023-01-01

**Authors:** Grigory S. Voronkov, Yana V. Aleksakina, Vladislav V. Ivanov, Aida G. Zakoyan, Ivan V. Stepanov, Elizaveta P. Grakhova, Muhammad A. Butt, Ruslan V. Kutluyarov

**Affiliations:** 1 Ufa University of Science and Technology, 32, Z. Validi St., 450076 Ufa, Russia; 2 Samara National Research University, 443086 Samara, Russia

**Keywords:** integrated photonics, silicon photonics, refractometry, gas sensing, optoelectronic oscillator, interrogation

## Abstract

Lab-on-a-chip systems are currently one of the most promising areas in the development of ultra-compact sensor systems, used primarily for gas and liquid analysis to determine the concentration of impurities. Integrated photonics is an ideal basis for designing “lab-on-a-chip” systems, advantageous for its compactness, energy efficiency, and low cost in mass production. This paper presents a solution for “lab-on-a-chip” device realization, consisting of a sensor and an interrogator based on a silicon-on-insulator (SOI) integrated photonics platform. The sensor function is performed by an all-pass microring resonator (MRR), installed as a notch filter in the feedback circuit of an optoelectronic oscillator based on an electro-optic phase modulator. This structure realizes the frequency interrogation of the sensor with high accuracy and speed using a conventional single-mode laser source. The system sensitivity for the considered gases is 13,000 GHz/RIU. The results show that the use of frequency interrogation makes it possible to increase the intrinsic *LoD* by five orders. The proposed solution opens an opportunity for fully integrated implementation of a photonic “laboratory-on-a-chip” unit.

## 1. Introduction

Gas detection and measurement of its concentration is currently an urgent task in ensuring the safety of residential and industrial buildings in environmental monitoring, industry, and medical diagnostics. In this case, gases that are most often the target of detection are those which are dangerous to humans either due to their toxicity or explosiveness, for example, carbon monoxide, ammonia, nitrous oxide, nitrous oxide, and sulfur dioxide [1]. The speed of the sensor reaction is important in industry, since hydrogen, for example, becomes explosive at a concentration of 4%, which requires a high rate of detection of gas in the air [2]. At low concentrations of acetone in the air, irritation of the skin and mucous membranes is observed, and an increase in this concentration can lead to the failure of the human central nervous system [3]. A similar set of gases is considered in monitoring the state of human health based on the composition of the exhaled gases [4], where the time of analysis of these gases plays a huge role in monitoring patient illness.

In power engineering, special attention is also paid to hydrogen and its compounds when monitoring the state of oil transformers [5,6,7]. Separately, it is also necessary to highlight the development of environmental monitoring systems [5], including the use of unmanned aerial vehicles [6,7]. In all described application situations, it is necessary to provide low sensor weight, small sensor dimensions, low power consumption, and high sensitivity. When used in explosive environments, it is additionally necessary to exclude the possibility of sparking as much as possible. These criteria are met by sensors based on photonics integrated circuits (PIC). They can be implemented in various structures, such as Mach–Zehnder interferometers [8,9,10], couplers [11,12], microring resonators (MRRs) [2,13,14], and more complex solutions, for example, MRRs based on subwavelength grating waveguides [15,16,17] and cascaded ring resonators [18,19]. Additionally, sensors can be implemented based on Bragg gratings [20], where the sensitivity can be increased by introducing slots; sensitivities are 113 nm/RIU for a conventional MRR sensor, about 594 nm/RIU for a single-slot MRR sensor, and about 708 nm/RIU for a two-slot MRR sensor [21].

In addition, coatings can be used in sensors, for example, polyhexamethylene biguanide (PHMB) [13,22], palladium [23,24], and zinc oxide [25,26]. It should be noted that the use of coatings in sensors based on integrated photonics raises questions about the possibility of their repeated use (the coatings can change properties over time) [14]. Therefore, for gas sensing, it seems expedient to study an MRR not covered with a functional layer.

Known methods of interrogation of optical sensors consist of estimating the shift of the resonant wavelength of the sensor, which depends on the value measured by the sensor [6,7,27]. Using an optical spectrum analyzer (OSA) is not the best solution due to the slow polling rate and high cost. These problems can be overcome by applying methods based on converting the wavelength of an optical sensor into a change in the parameters of the electrical signal (power, frequency, and signal envelope). An analysis of the methods showed that the frequency domain conversion shows the best results in terms of interrogation rate and resolution [28], which is important due to small changes in the refractive index (RI) of the studied gases. However, this article discusses a method based on the use of an optoelectronic oscillator (OEO) [29]. The value of its microwave frequency is determined by the resonant wavelength of the optical device used to detect the gas composition. It should be noted that a notch filter is a necessary element to start the OEO generation. This filter, in interrogation systems, is a sensitive element. This method effectively transfers the measurement from the optical to the electrical domain, thus providing a much higher frequency resolution, making very high measurement resolution possible. Additionally, this method can provide a high interrogation rate, since the microwave signal can be measured by a DAC at high speed and high resolution. In several works [30,31,32,33,34,35,36], OEO-based interrogation systems have been proposed that offer various ways to implement an OEO-based optical sensor, i.e., a microwave photonic filter. These include phase-shifted fiber Bragg gratings (PS-FBG) and polarization-maintaining PS-FBG [30,32], Mach–Zehnder fiber interferometers [31], and microcavities [36]. Note that the presented optical devices have the frequency characteristics of a notch filter; the change in its stop frequency reflects the changes in the environment (gas composition, temperature, humidity, etc.).

This work aims to design and analyze a sensing system based on integrated photonics with frequency interrogation, using an all-pass MRR as a sensitive element. The integrated photonic MRR is a key element that makes it possible to realize highly sensitive measurements for gas identification. In our earlier work [37], we showed that implementing a fully integrated sensor system using microring resonators is associated with finding a trade-off between sensitivity and a dynamic range of measurements, while also considering the source radiation’s bandwidth. As we have shown, this problem can be partly solved by using an OEO in the intensity interrogation scheme as a signal source. However, this approach only requires the use of a MCR add-drop. At the same time, an add-drop waveguide introduces additional losses in the MCR and switches it to the under coupling mode, which reduces the sensitivity [38]. A more detailed comparison of all-pass and add-drop sensors as elements of a gas detection sensor system is given in Section 3. The proposed system, due to the integrated implementation on the SOI platform, simultaneously meets the requirements for the overall dimensions of sensors and at the same time provides a significant increase in sensitivity. In this paper, the proposed system is used to measure gas compositions. Due to the high sensitivity of the SOI-based ring resonator, a small change in gas composition or other parameters results in a shift in the resonant frequency of the sensing element, which can be detected by monitoring the oscillatory radio frequency peak at the output of the optoelectronic oscillator (OEO). In particular, SOI μ-resonators have shown that they can effectively respond to changes in gas composition [13,39,40,41].

The relatively large overall dimensions of the bulk optical components (both active and passive) that make up an OEO system present a significant challenge, especially for sensor applications where compactness, portability, and light weight are critical criteria for use in measuring environmental variables. Integrated photonic technologies can solve this problem due to their advantages in small size, weight, and power consumption, which makes them attractive compared to bulky fiberoptic components to meet the increasingly demanding requirements of sensors [42].

## 2. Sensing System Principle and Design

**Figure 1:**
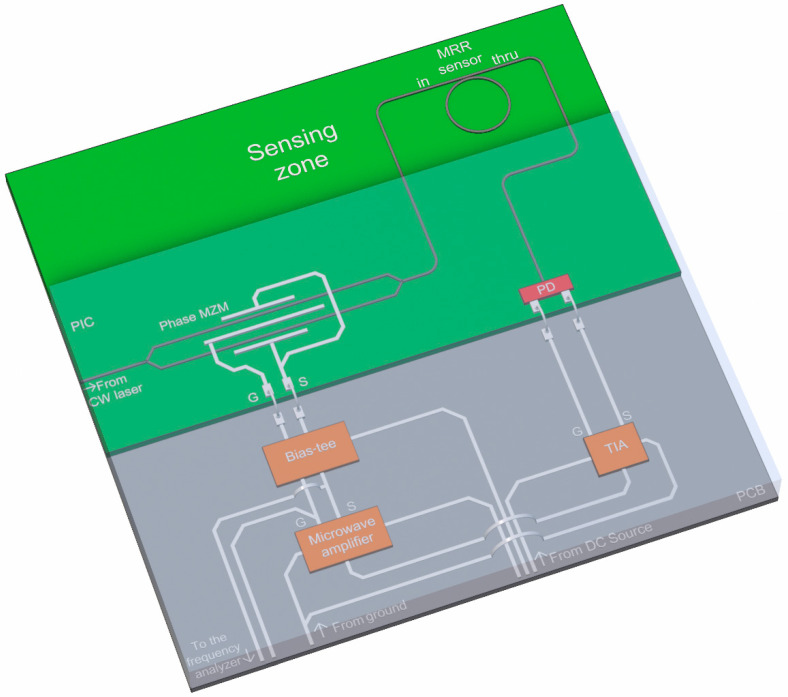
Sensing system structure and layout (not to scale).

Figure 1 shows the scheme of the gas sensing system. The all-pass MRR acts as a sensitive element and is housed in the feedback path of an interrogator. The interrogator is represented by an OEO based on a phase Mach–Zehnder modulator (MZM). A shift in the MRR’s resonant wavelength associated with the refractive index variation of its environment causes a frequency change in the OEO output signal. In addition to the notch filter described, the feedback path includes a photodetector (PD), a trans-impedance amplifier (TIA), and a microwave amplifier.

The operating principle of OEO is as follows. The narrowband signal from the CW (continuous wave) laser is fed to the phase MZM and is initially modulated by the noise generated by the photodetector. The most important factor is that the modulated signal’s spectral components are symmetric with the carrier and antiphase. Supplying such a signal to the photodiode leads to mutual suppression of the antiphase components, resulting in zero microwave signal at the OEO output. The bandpass filter suppresses the modulated signal’s narrow spectral component, which causes an uncompensated optical signal at the detector input with a wavelength determined by the mismatch between the CW laser carrier frequency and the resonant frequency of the notch filter (MRR). Of fundamental importance is the presence of resonance in the MRR. Let us consider the output signal from MZM, *u_PM_*(*t*), which is modulated by the signal, *s*_Ω_(*t*):

(1) $$u_{PM}(t)=U_{0}\cos(\omega_{0}t+Ms_{\Omega }(t)).$$

where *ω*_0_ is the angular frequency of the carrier, *U*_0_ is its amplitude, and *M* is the modulation index. Let us assume that the signal, *s*_Ω_(*t*), has a unit amplitude and it is represented as a set of harmonic oscillations:

(2) $$s_{\Omega }\left(t\right)=\sum_{i=1}^{n}\sin\omega_{i}t.$$

Then, the modulated signal is written as:

(3) $$u_{PM}\left(t\right)=U_{0}\cos\left(\omega_{0}t+M\sum_{i=1}^{n}\sin\omega_{i}t\right),$$

(4) uPMt=U0Reexpjω0texpjM∑i=1∞sinωit.

Then, using the known representation:

(5) $$\exp\left(jM\sin x\right)=\sum_{k=-\infty }^{\infty }J_{k}\left(M\right)\exp\left(jkx\right),$$

where $J_{k}\left(M\right)$ is the k-order Bessel function of the argument *M*, we get:

(6) uPMt=U0Reexpjω0t∑k=−∞∞JkMexpjk∑i=1nsinωit.

**Figure 2:**
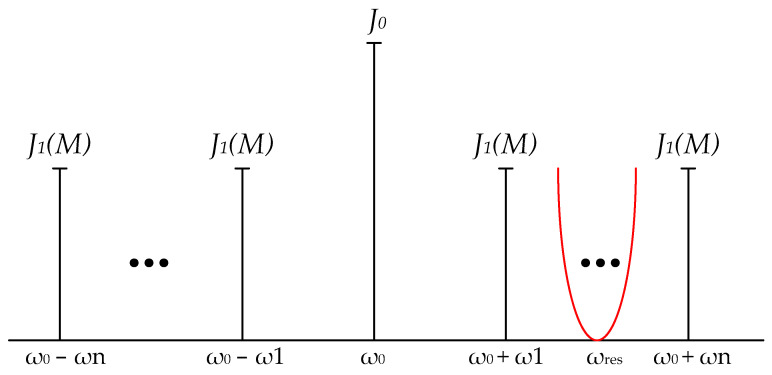
OEO spectrum and generating principle.

If we neglect the high orders of the Bessel functions ($\left|k\right|>1$, so we take it into account only $J_{1}\left(M\right)$), the signal spectrum at the output of the phase modulator has the shape presented in Figure 2. The red line conventionally shows the stopband of the MRR notch filter with the resonance frequency *ω_res_*.

The signal sidebands are limited by the frequencies ω_0_ + ω_1_, ω_0_ + ω_n_, ω_0_−ω_1_, and ω_0_−ω_n_. MRR suppresses part of the spectrum of the upper band of the phase-modulated signal. Thus, an unsuppressed symmetric band does not have antiphase components on the photodetector. Consequently, only the carrier and a signal with a frequency of ω_0_−ω_res_ are present at the photodetector, resulting in a narrowband microwave oscillation. Therefore, the spectrum of optical signals that cause microwave generation is also narrow. Thus, the signal bandwidth at the thru-port of the sensor is more limited than the bandpass filter’s *FWHM* (full width at half maximum). This increases the system quality relative to the single resonator, which upgrades the sensitivity and resolution.

## 3. MRR-Based Sensor Design and Simulation

The main requirement for the sensor design was a small footprint while maintaining a high quality and sensitivity, as well as a simple structure. The gases of interest in terms of the above analysis are shown in Table 1.

As mentioned above, it is more challenging to achieve the boundary coupling conditions for add-drop MRR than for all-pass. Below is a comparison of all-pass and add-drop sensors calculated using the FDTD (finite difference time domain) method using Ansys Lumerical software. MRR radius and gap were chosen to provide ring waveguide transmission and a coupling coefficient close to the critical coupling regime [38]. We calculated the sensors’ parameters for the TE-mode at the resonant wavelengths and the numerically optimized waveguide width and height to improve TE-mode transmission and excitation [48] and to accommodate common manufacturing platforms [49]. The RI of Si is equal to 3.4955 [50].

### 3.1. Add-Drop MRR

Table 2 shows the dimensions of the add-drop MRR sensor. The MRR resonant wavelength in a vacuum is equal to 1545.75 nm.

**Figure 3:**
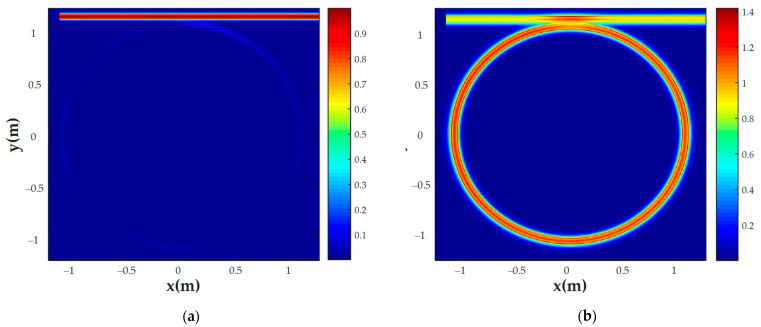
E-field distribution of the CCl_4_ sensor at (**a**) a non-resonant wavelength (1532.39 nm) and (**b**) a resonant wavelength (1543.2 nm).

**Figure 4:**
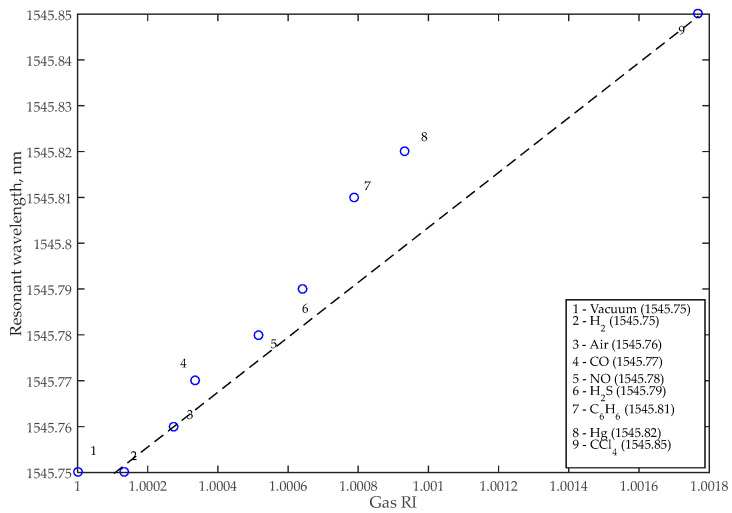
Relationship between ambient refractive index and resonant wavelength for the add-drop sensor.

MRR transmission and coupling coefficients were also calculated using the FDTD method. The gap between the waveguides and the ring was set to 0.16 μm. As mentioned above, these values were found using numerically calculated MRR waveguide transmissions to fulfill the critical coupling criterion [38]. The E-field distributions in the non-resonant state and resonant state are shown in Figure 3a,b, respectively. The resonant properties of the MRR were estimated based on the quality factor (*Q-factor*), full width at half maximum (*FWHM*), and sensitivity (*S*):

(7) $$Q-factor=\frac{\lambda_{res}}{FWHM};$$

(8) $$FWHM=\frac{\left(1-r^{2}a\right)\lambda_{res}^{2}}{\pi n_{g}L\sqrt{r^{2}a}};$$

(9) $$S=\left|\frac{\lambda_{CCl_{4}}-\lambda_{air}}{n_{CCl_{4}}-n_{air}}\right|,$$

where *a* = exp(α*L*/2) is the MRR transmission measured in FDTD; α is the MRR waveguide attenuation coefficient; *r* is the coupler transmission to the in-through waveguide; λ*_res_* is the MRR resonant wavelength (λ*_res_* = *Ln_eff_*/*m*, where positive integer *m* is the resonance number); *L* is the MRR circumference; *n_g_* and *n_eff_* are the numerically counted values of the waveguide group and effective refractive indices, respectively; $\lambda_{CCl_{4}}$ and $\lambda_{air}$ are the resonant wavelengths of the gas with maximum RI (CCl_4_) and the reference gas (air), respectively; and $n_{CCl_{4}}$ and $n_{air}$ are the RI of the CCl_4_ gas and the reference gas (air), respectively. We should also note that the sensitivity, *S*, may also be determined as the slope of the curve in Figure 4, equal to 56 nm/RIU.

The intrinsic limit of detection (*LoD*) is usually calculated to estimate the sensor quality. It can also be obtained as follows [51]:

(10) $$LoD(n)=\frac{\lambda_{res}}{S\cdot Q-factor}.$$

**Figure 5:**
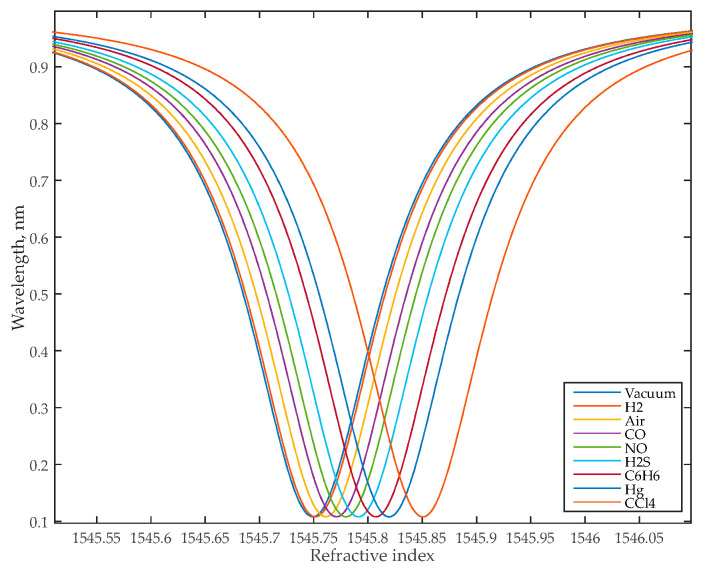
Transmission spectra of the add-drop MRR sensor for different ambient gases.

**Figure 6:**
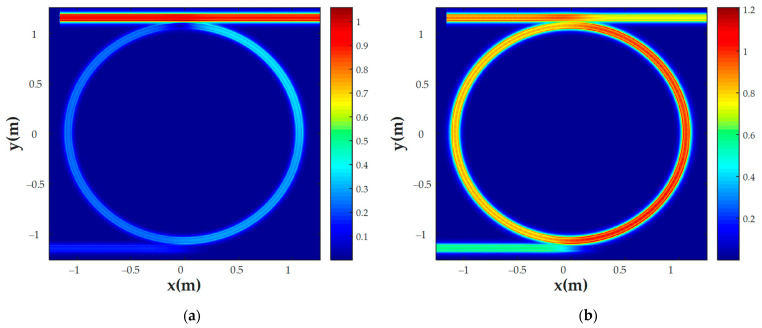
E-field distribution of the CCl_4_ sensor at (**a**) a non-resonant wavelength (1545.03 nm) and (**b**) a resonant wavelength (1545.73 nm).

The sensor was simulated using the finite element method (FEM) and then imported into the INTERCONNECT modeling environment. INTERCONNECT is a part of the Ansys Lumerical software tool designed for PIC system modeling. To simulate MRR in INTERCONNECT we calculated its parameters (transmission, coupling effective RI, and group RI) in Lumerical using the finite-difference time-domain (FDTD) and FEM solvers. As a result of the calculations, the dependencies of the transmission spectrum of the MRR on the wavelength (Figure 5) were obtained for various gaseous substances that are odorless but dangerous to human health. Figure 6 shows the E-field distribution in the MRR for CCl_4_ analysis, and Table 3 shows the add-drop sensor’s characteristics.

### 3.2. All-Pass MRR

**Figure 7:**
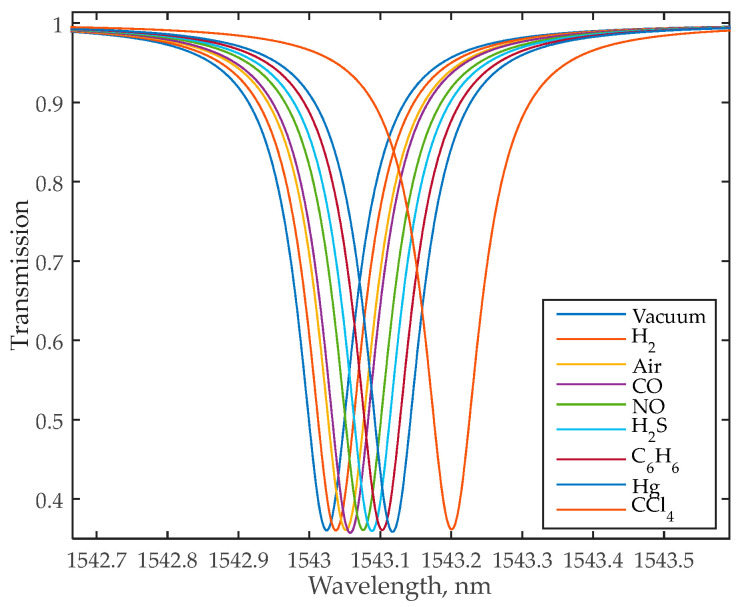
Transmission spectrum of the MRR for different ambient gases.

**Figure 8:**
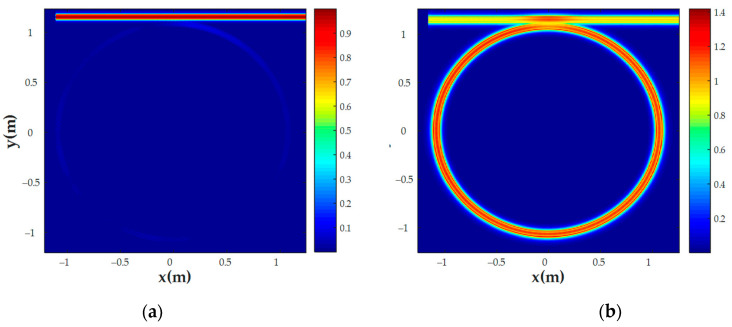
E-field distribution of the CCl_4_ sensor at (**a**) a non-resonant wavelength (1532.39 nm) and (**b**) a resonant wavelength (1543.2 nm).

We performed the same simulations for the all-pass MRR sensor with the same geometric parameters as described in Section 3.1. Figure 7 shows the transmission spectra of this sensor for different gases, Figure 8 shows the E-field distribution in the MRR for CCl_4_ analysis, and Table 4 lists the all-pass sensor characteristics for different gases.

One can see that applying the all-pass sensor will improve the system sensitivity from 60 nm/RIU to 100 nm/RIU, which corresponds to the sensitivity of the conventional MRR sensor given in [21], and the intrinsic *LoD* decreases from 23.34⋅10^−4^ RIU to 9⋅10^−4^. Therefore, the all-pass sensor is preferable for use in a sensing system with frequency interrogation.

## 4. Sensing System Simulation and Performance Analysis

**Figure 9:**
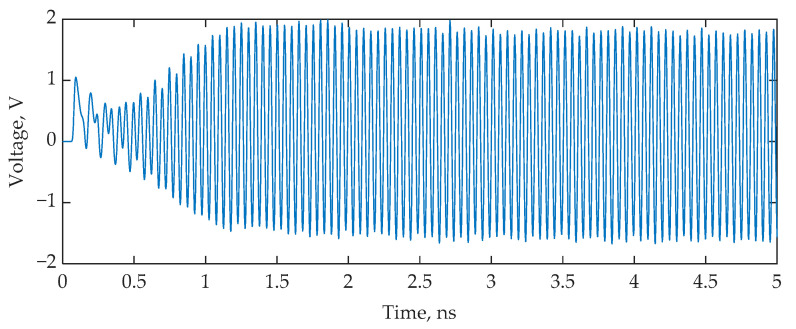
OEO start process for SOI-based MRR interrogation.

**Figure 10:**
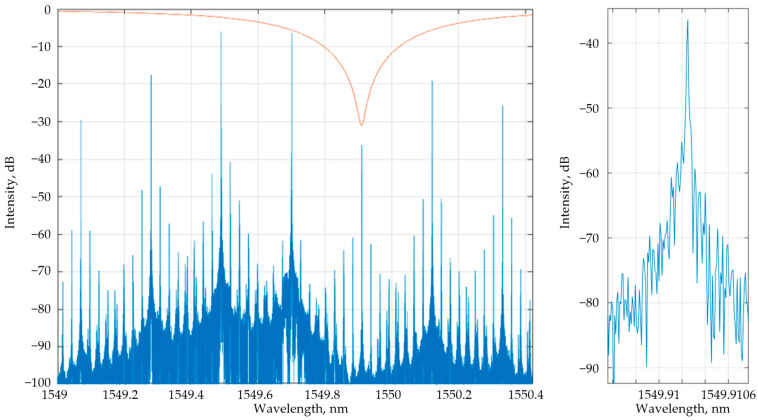
Transmission spectra at the thru-port for CO detection using an SOI-based MRR.

**Figure 11:**
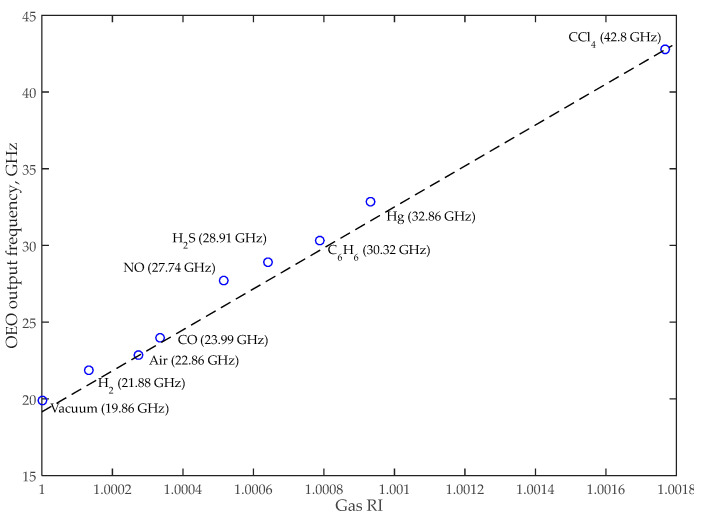
OEO output frequency dependence on gas RI using an SOI-based MRR.

A simulation was carried out, following the scheme shown in Figure 1, in the Lumerical Interconnect environment with the following parameters. The CW laser was set to 1549.7 nm with an output power of 2 mW. A typical SOI-based structure [52] was applied to the PD numerical model. The PD dark current was 60 nA; the PD, amplifier, and MZM RF bandwidths were equal to 50 GHz; and the MZM *V_π_* = 4 V. To initiate generation, microwave amplifiers with a total gain of 65 dB were used. Figure 9 shows the OEO timing diagram. The trigger transient takes less than 1.5 ns, which can provide a sampling rate of more than 600 MHz. Figure 10 shows the spectrum at the output of the sensor’s thru-port and the thru-port transfer function for CO detection. The bandwidth of the optical radiation is much smaller than the *FWHM* of the sensor, which confirms the hypothesis presented in Section 2 that the quality factor of the sensor system is increased due to frequency interrogation. Figure 11 demonstrates the dependence of the OEO output frequency on the refractive index of the gases.

We compared the *LoD* parameters to verify the hypothesis on the possibility of improving sensor parameters during interrogation by the frequency method. The comparison results are shown in Table 5.

The sensitivity of the sensing system, $S_{ss}$, was calculated as follows:

(11) $$S_{ss}=\left|\frac{f_{g}-f_{air}}{n_{g}-n_{air}}\right|,$$

where *f*_g_ and *f_air_* are the resonant wavelengths of the selected gas and the reference gas (*air*), respectively, and *n_g_* and *n_air_* are the RI of the selected gas and the reference gas (*air*), respectively.

## 5. Discussion

The paper describes an approach to improving the performance of a sensing system’s by applying frequency interrogation. Although the simulation results confirm the proposed method’s effectiveness, it has several limitations. The startup speed of OEO is largely determined by the gain of the microwave amplifier in the feedback circuit, which can negatively affect the energy efficiency of the sensor system. Another limitation is the frequency selectivity of the microwave circuit. With a microwave circuit working bandwidth of 50 GHz, the largest difference between the optical carrier wavelengths and the notch filter the system can detect will be approximately 400 pm. On the other hand, such a limitation makes it possible to confidently use resonant structures with an FSR greater than 800 pm as notch filters (this criterion is met, for example, by MRR at SOI).

Another problem of the considered scheme is the need to measure the microwave frequency in a wide range. The output frequency of the OEO can be reduced by bringing the optical source wavelength closer to the resonant wavelength of the sensor. However, this will lower the optical carrier level. These losses can be compensated for by increasing the gain of the microwave amplifier, but this can lead to an increase in system noise, device temperature, and overall power consumption.

The third problem to be solved in the future is the temperature dependence of resonant structures. According to [53], SOI-based resonant structures are temperature-dependent. Therefore, the sensing system should be thermostable. Another solution to this problem is to apply machine-learning algorithms to improve sensing system quality [54].

At the same time, the described sensing system has several advantages. First of all, frequency interrogation allows the use of more sensitive sensors. Secondly, considering Figure 10, applying OEO decreases the sensor’s *FWHM* by about 3 orders of magnitude. Numerically it leads to a rise in the Q-factor and a decrease in *LoD*, according to Equations (7) and (11). However, it is hard to estimate the system *LoD* using only Equation (11) because it does not take into account the element’s noise. At the same time, if we consider the system *LoD* as the minimum frequency change that the system can detect, we may try to estimate *LoD* as follows. The resonant wavelength, $\lambda_{airres}$, of MRR for the air medium is equal to 1543.05 nm (see Table 4). If we consider that the minimum microwave frequency shift, Δf, that can be measured equals 100 kHz (usually in microwave systems the frequency stability is higher [55]), then we can estimate the equivalent wavelength shift, Δλ, as follows:

(12) $$\Delta \lambda =\left|\lambda_{airres}-\frac{c}{\frac{c}{\lambda_{airres}}+\Delta f}\right|=7.94\cdot 10^{-7}\;\mathrm{nm}.$$

Using this value we may find the *LoD* using the following equation [56]:

(13) $$LoD=\frac{\Delta \lambda }{S}=7.94\cdot 10^{-9}\;\mathrm{RIU}.$$

Therefore, applying the frequency interrogation method has resulted in a decrease in the *LoD* of 5 orders of magnitude.

## 6. Conclusions

The paper presents a simulation of a sensor system based on integrated photonics for gas detection using the SOI platform. We chose the sensor’s dimensions to accommodate for common manufacturing platforms. The sensor’s sensitivity equals 100 nm/RIU, estimated from the numerical simulation results of the detection of various gases. Sensor scanning was performed using a frequency interrogator on an MZM-based OEO. This design means that most parts of the described system (the sensor, phase MZM, and photodiode) are a single PIC, keeping only the CW source and the microwave amplifier as discrete elements. It also allows the implementation of a more sensitive all-pass MRR sensor. The system sensitivity for the considered gases is 13,000 GHz/RIU. The results show that the use of frequency interrogation makes it possible to increase the *LoD* by at least two-fold (up to five-fold for some gases).

## Figures and Tables

**Table 1:** Refractive indices of gases under the analysis.

Gas	Chemical Formula	*n* (*RI*)
Vacuum	-	1.0
Hydrogen	H_2_	1.000132 [43]
Air	-	1.000273 [44]
Carbon monoxide	CO	1.000334 [45]
Nitric oxide	NO	1.000516 [45]
Hydrogen sulfide	H_2_S	1.000641 [46]
Benzene	C_6_H_6_	1.000788 [46]
Mercury vapor	Hg	1.000933 [47]
Carbon tetrachloride	CCl_4_	1.001768 [46]

**Table 2:** Add-drop MRR sensor parameters.

Parameter	Value
Outer radius	11 μm
Waveguide height	0.22 μm
Waveguide width	0.4 μm
Gap	0.16 μm
Ambient medium	Vacuum (*n* = 1.0)

**Table 3:** Add-drop sensor characteristics for different gases.

Gas	Chemical Formula	*n*	Resonant Wavelength, nm	FWHM, pm	Sensitivity, nm/RIU	Q-Factor	LOD, 10^−4^ RIU
Vacuum	-	1.0	1545.74	140	56	11,040	23.34
Hydrogen	H_2_	1.00013	1545.75				
Air	-	1.000273	1545.76				
Carbon monoxide	CO	1.000334	1545.77				
Nitric oxide	NO	1.000516	1545.78				
Hydrogen sulfide	H_2_S	1.000641	1545.79				
Benzene	C_6_H_6_	1.000788	1545.81				
Mercury vapor	Hg	1.000933	1545.82				
Carbon tetrachloride	CCl_4_	1.001768	1545.85				

**Table 4:** All-pass sensor characteristics for different gases.

Gas	Chemical Formula	*n*	Resonant Wavelength, nm	FWHM, pm	Sensitivity, nm/RIU	Q-Factor	LOD, 10^−4^ RIU
Vacuum	-	1.0	1543.02	140	100	17,140	9
Hydrogen	H_2_	1.00013	1543.04				
Air	-	1.000273	1543.05				
Carbon monoxide	CO	1.000334	1543.06				
Nitric oxide	NO	1.000516	1543.07				
Hydrogen sulfide	H_2_S	1.000641	1543.09				
Benzene	C_6_H_6_	1.000788	1543.10				
Mercury vapor	Hg	1.000933	1543.12				
Carbon tetrachloride	CCl_4_	1.001768	1543.20				

**Table 5:** System sensitivity for frequency interrogation.

Gas	Chemical Formula	*n*	System Sensitivity, GHz/RIU
Vacuum	-	1.0	13,000
Hydrogen	H_2_	1.000132	
Carbon monoxide	CO	1.000334	
Nitric oxide	NO	1.000516	
Hydrogen sulfide	H_2_S	1.000641	
Benzene	C_6_H_6_	1.000788	
Mercury vapor	Hg	1.000933	
Carbon tetrachloride	CCl_4_	1.001768	

## Data Availability

Not applicable.

## References

[1] Wang H., Ma J., Zhang J., Feng Y., Vijjapu M.T., Yuvaraja S., Surya S.G., Salama K.N., Dong C., Wang Y. (2021). Gas Sensing Materials Roadmap. J. Phys. Condens. Matter.

[2] Noh J.-S., Lee J.M., Lee W. (2011). Low-Dimensional Palladium Nanostructures for Fast and Reliable Hydrogen Gas Detection. Sensors.

[3] Yang G., Zhang M., Dong D., Pan X., Zhou Y., Han S.-T., Xu Z., Wang W., Yan Y. (2019). TiO _2_ Based Sensor with Butterfly Wing Configurations for Fast Acetone Detection at Room Temperature. J. Mater. Chem. C.

[4] Tai H., Wang S., Duan Z., Jiang Y. (2020). Evolution of Breath Analysis Based on Humidity and Gas Sensors: Potential and Challenges. Sens. Actuators B Chem..

[5] Butt M.A., Voronkov G.S., Grakhova E.P., Kutluyarov R.V., Kazanskiy N.L., Khonina S.N. (2022). Environmental Monitoring: A Comprehensive Review on Optical Waveguide and Fiber-Based Sensors. Biosensors.

[6] Wang Y., Li Y., Liao C., Wang D.N., Yang M., Lu P. (2010). High-Temperature Sensing Using Miniaturized Fiber In-Line Mach–Zehnder Interferometer. IEEE Photon. Technol. Lett..

[7] Xu H., Hafezi M., Fan J., Migdall A., Strouse G., Ahmed Z., Taylor J.M. (2013). Photonic Temperature Sensor Based on Microring Resonators. Proceedings of the CLEO: 2013.

[8] El Shamy R.S., Khalil D., Swillam M.A. (2020). Mid Infrared Optical Gas Sensor Using Plasmonic Mach-Zehnder Interferometer. Sci Rep.

[9] Fabricius N., Gauglitz G., Ingenhoff J. (1992). A Gas Sensor Based on an Integrated Optical Mach-Zehnder Interferometer. Sensors and Actuators B Chem..

[10] El Shamy R.S., Swillam M.A., ElRayany M.M., Sultan A., Li X. (2021). Compact Gas Sensor Using Silicon-on-Insulator Loop-Terminated Mach–Zehnder Interferometer. Photonics.

[11] Sun L., Semenova Y., Wu Q., Liu D., Yuan J., Ma T., Sang X., Yan B., Wang K., Yu C. (2017). High Sensitivity Ammonia Gas Sensor Based on a Silica-Gel-Coated Microfiber Coupler. J. Light. Technol..

[12] Li D., Wu G., Chen J., Yan S., Liu Z., Xu F., Lu Y. (2018). Ethanol Gas Sensor Based on a Hybrid Polymethyl Methacrylate–Silica Microfiber Coupler. J. Light. Technol..

[13] Mi G., Horvath C., Aktary M., Van V. (2016). Silicon Microring Refractometric Sensor for Atmospheric CO_2_ Gas Monitoring. Opt. Express.

[14] Mi G., Horvath C., Van V. (2017). Silicon Photonic Dual-Gas Sensor for H_2_ and CO_2_ Detection. Opt. Express.

[15] Yan H., Huang L., Xu X., Chakravarty S., Tang N., Tian H., Chen R.T. (2016). Unique Surface Sensing Property and Enhanced Sensitivity in Microring Resonator Biosensors Based on Subwavelength Grating Waveguides. Opt. Express.

[16] Tu Z., Gao D., Zhang M., Zhang D. (2017). High-Sensitivity Complex Refractive Index Sensing Based on Fano Resonance in the Subwavelength Grating Waveguide Micro-Ring Resonator. Opt. Express.

[17] Xu Y., Fu C., Sun S., Kong M. (2022). Wide-Range Refractive Index Sensing Relied on Tracking the Envelope Spectrum of a Dispersive Subwavelength Grating Microring Resonator. Optics Laser Technol..

[18] Liu Y., Li Y., Li M., He J.-J. (2017). High-Sensitivity and Wide-Range Optical Sensor Based on Three Cascaded Ring Resonators. Opt. Express.

[19] Jin L., Li M., He J.-J. (2011). Highly-Sensitive Silicon-on-Insulator Sensor Based on Two Cascaded Micro-Ring Resonators with Vernier Effect. Optics Commun..

[20] Liu C., Sang C., Wu X., Cai J., Wang J. (2021). Grating Double-Slot Micro-Ring Resonator for Sensing. Optics Commun..

[21] Yuan G., Gao L., Chen Y., Liu X., Wang J., Wang Z. (2014). Improvement of Optical Sensing Performances of a Double-Slot-Waveguide-Based Ring Resonator Sensor on Silicon-on-Insulator Platform. Optik.

[22] Butt M.A., Kazanskiy N.L., Khonina S.N. (2022). On-Chip Symmetrically and Asymmetrically Transformed Plasmonic Bragg Grating Formation Loaded with a Functional Polymer for Filtering and CO_2_ Gas Sensing Applications. Measurement.

[23] Das C., Mohammad Z., Alam M.M. Optical Hydrogen Gas Sensor Based on Palladium Coated Microring Resonator. Proceedings of the 2018 International Conference on Innovation in Engineering and Technology (ICIET).

[24] Eryürek M., Karadag Y., Taşaltın N., Kılınç N., Kiraz A. (2015). Optical Sensor for Hydrogen Gas Based on a Palladium-Coated Polymer Microresonator. Sens. Actuators B Chem..

[25] Yebo N.A., Lommens P., Hens Z., Baets R. (2010). An Integrated Optic Ethanol Vapor Sensor Based on a Silicon-on-Insulator Microring Resonator Coated with a Porous ZnO Film. Opt. Express.

[26] Zhang X., Li Z., Sun Y., Tong K. (2014). Simulation of ZnO-Coated SOI Microring Resonant Shift Response to Ethanol and Ammonia. Optik.

[27] Yao J. (2021). Microwave Photonic Sensors. J. Light. Technol..

[28] Yao X.S., Maleki L. (1996). Optoelectronic Microwave Oscillator. J. Opt. Soc. Am. B.

[29] Li W., Yao J. (2010). An Optically Tunable Optoelectronic Oscillator. J. Light. Technol..

[30] Wang Y., Zhang J., Yao J. (2016). An Optoelectronic Oscillator for High Sensitivity Temperature Sensing. IEEE Photon. Technol. Lett..

[31] Zou X., Liu X., Li W., Li P., Pan W., Yan L., Shao L. (2016). Optoelectronic Oscillators (OEOs) to Sensing, Measurement, and Detection. IEEE J. Quantum Electron..

[32] Zhang S., Chen H., Fu H. Fiber-Optic Temperature Sensor Using an Optoelectronic Oscillator. Proceedings of the 2015 14th International Conference on Optical Communications and Networks (ICOCN).

[33] Zhu Y., Jin X., Chi H., Zheng S., Zhang X. (2014). High-Sensitivity Temperature Sensor Based on an Optoelectronic Oscillator. Appl. Opt..

[34] Saleh K., Bouchier A., Merrer P.H., Llopis O., Cibiel G. Fiber Ring Resonator Based Opto-Electronic Oscillator: Phase Noise Optimisation and Thermal Stability Study. Proceedings of the SPIE 7936, RF and Millimeter-Wave Photonics.

[35] Bogaerts W., De Heyn P., Van Vaerenbergh T., De Vos K., Kumar Selvaraja S., Claes T., Dumon P., Bienstman P., Van Thourhout D., Baets R. (2012). Silicon Microring Resonators. Laser Photon. Rev..

[36] Chew S.X., Yi X., Yang W., Wu C., Li L., Nguyen L., Minasian R. (2017). Optoelectronic Oscillator Based Sensor Using an On-Chip Sensing Probe. IEEE Photonics J..

[37] Voronkov G., Zakoyan A., Ivanov V., Iraev D., Stepanov I., Yuldashev R., Grakhova E., Lyubopytov V., Morozov O., Kutluyarov R. (2022). Design and Modeling of a Fully Integrated Microring-Based Photonic Sensing System for Liquid Refractometry. Sensors.

[38] Milvich J., Kohler D., Freude W., Koos C. (2021). Integrated Phase-Sensitive Photonic Sensors: A System Design Tutorial. Adv. Opt. Photon..

[39] Guha P., Ali S., Lee C., Udrea F., Milne W., Iwaki T., Covington J., Gardner J. (2007). Novel Design and Characterisation of SOI CMOS Micro-Hotplates for High Temperature Gas Sensors. Sens. Actuators B Chem..

[40] Samotaev N., Pisliakov A., Filipchuk D., Etrekova M., Biro F., Ducso C., Bársony I., Velichko E., Vinnichenko M., Kapralova V., Koucheryavy Y. (2021). SOI Based Micro-Bead Catalytic Gas Sensor. International Youth Conference on Electronics, Telecommunications and Information Technologies.

[41] Lu C.-C., Liao K.-H., Udrea F., Covington J.A., Gardner J.W. (2008). Multi-Field Simulations and Characterization of CMOS-MEMS High-Temperature Smart Gas Sensors Based on SOI Technology. J. Micromech. Microeng..

[42] Koushik K.P., Malathi S., Kadambi G.R., Kumar P.B., Palade V. (2020). Optical Micro-ring Resonator for Detection of Carbon Dioxide Gas. Emerging Trends in Photonics, Signal Processing and Communication Engineering.

[43] Peck E.R., Huang S. (1977). Refractivity and Dispersion of Hydrogen in the Visible and Near Infrared. J. Opt. Soc. Am..

[44] Ciddor P.E. (1996). Refractive Index of Air: New Equations for the Visible and Near Infrared. Appl. Opt..

[45] Smith P.L., Huber M.C.E., Parkinson W.H. (1976). Refractivities of H_2_, He, O_2_, CO, and Kr for 168 ≤ λ ≤ 288 nm. Phys. Rev. A.

[46] Moutzouris K., Papamichael M., Betsis S.C., Stavrakas I., Hloupis G., Triantis D. (2014). Refractive, Dispersive and Thermo-Optic Properties of Twelve Organic Solvents in the Visible and Near-Infrared. Appl. Phys. B.

[47] Inagaki T., Arakawa E.T., Williams M.W. (1981). Optical Properties of Liquid Mercury. Phys. Rev. B.

[48] Chrostowski L., Hochberg M.E. (2015). Silicon Photonics Design.

[49] Rahim A., Goyvaerts J., Szelag B., Fedeli J.-M., Absil P., Aalto T., Harjanne M., Littlejohns C., Reed G., Winzer G. (2019). Open-Access Silicon Photonics Platforms in Europe. IEEE J. Select. Topics Quantum Electron..

[50] Salzberg C.D., Villa J.J. (1957). Infrared Refractive Indexes of Silicon Germanium and Modified Selenium Glass. J. Opt. Soc. Am..

[51] Wang X., Flueckiger J., Schmidt S., Grist S., Fard S.T., Kirk J., Doerfler M., Cheung K.C., Ratner D.M., Chrostowski L. (2013). A Silicon Photonic Biosensor Using Phase-Shifted Bragg Gratings in Slot Waveguide. J. Biophoton..

[52] Liow T.Y., Ang K.W., Fang Q., Song J.F., Xiong Y.Z., Yu M.B., Lo G.Q., Kwong D.L. (2010). Silicon Modulators and Germanium Photodetectors on SOI: Monolithic Integration, Compatibility, and Performance Optimization. IEEE J. Select. Topics Quantum Electron..

[53] Sana A.K., Jun M., Yokoyama S., Amemiya Y., Yokoyama S. (2017). Temperature Dependence of Resonance Characteristics of Silicon Resonators and Thermal Stability Improvement by Differential Operation Method. Jpn. J. Appl. Phys..

[54] Tian X., Gunawan G., Zhou L., Li L., Nguyen L., Minasian R., Yi X. (2022). Athermal Microwave Photonic Sensor Based on Single Microring Resonance Assisted by Machine Learning. J. Light. Technol..

[55] Zou X., Lu B., Pan W., Yan L., Stöhr A., Yao J. (2016). Photonics for Microwave Measurements: Photonics for Microwave Measurements. Laser Photonics Rev..

[56] Zhao C.Y., Zhang L., Zhang C.M. (2018). Compact SOI Optimized Slot Microring Coupled Phase-Shifted Bragg Grating Resonator for Sensing. Optics Commun..

